# Supplementary material for: Enteroviral epitope mimicry enables NK cell-mediated targeting of ASPH in hepatocellular carcinoma
Source: bioRxiv. 2026 Apr 10:2026.04.07.717032. Preprint. [Version 1] doi: 10.64898/2026.04.07.717032 (PMC13082011; doi:10.64898/2026.04.07.717032)
Supplement: Supplement 1 [file media-1.pdf]

Supplemental Data for

Enteroviral epitope mimicry enables NK cell-mediated targeting of ASPH in hepatocellular carcinoma

Man Hsin Hung, Qin Li, Limin Wang, Marshonna Forgues, Andrew S. Lee, Lisa M. Jenkins, Tapan K. Maity, Jesse Buffington, Jittiporn Chaisaingmongkol, Siritida Rabibhadana, Mathuros Ruchirawat, Mitchell Ho, Xin Wei Wang

Correspondence to: xw3u@nih.gov (X.W.W.)

This material file includes:

Extended Data Figure 1 to 7

Extended Data Table 1

Extended Data Figure 1

A

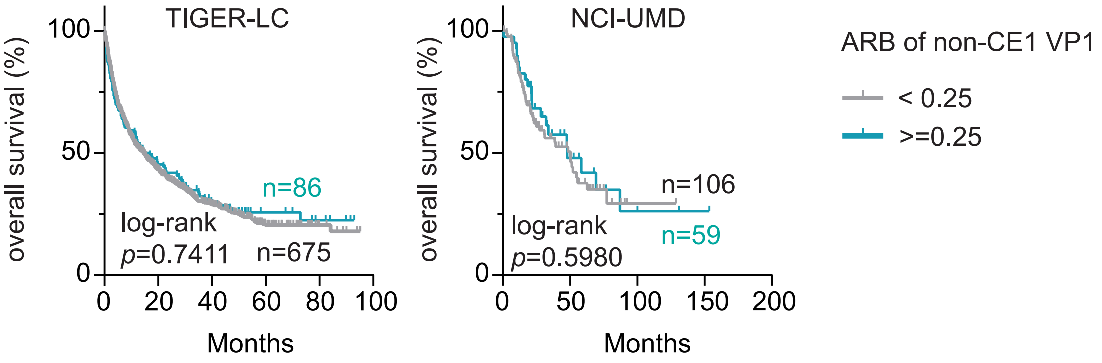

B

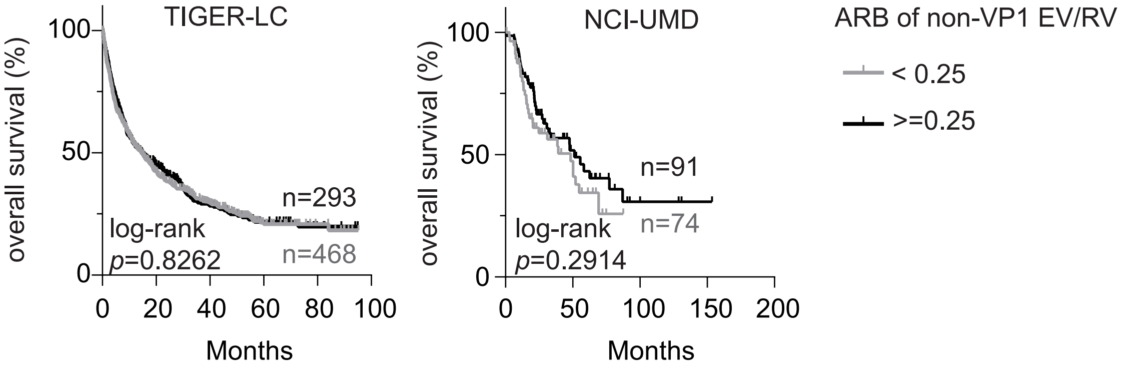

Extended Data Figure 2

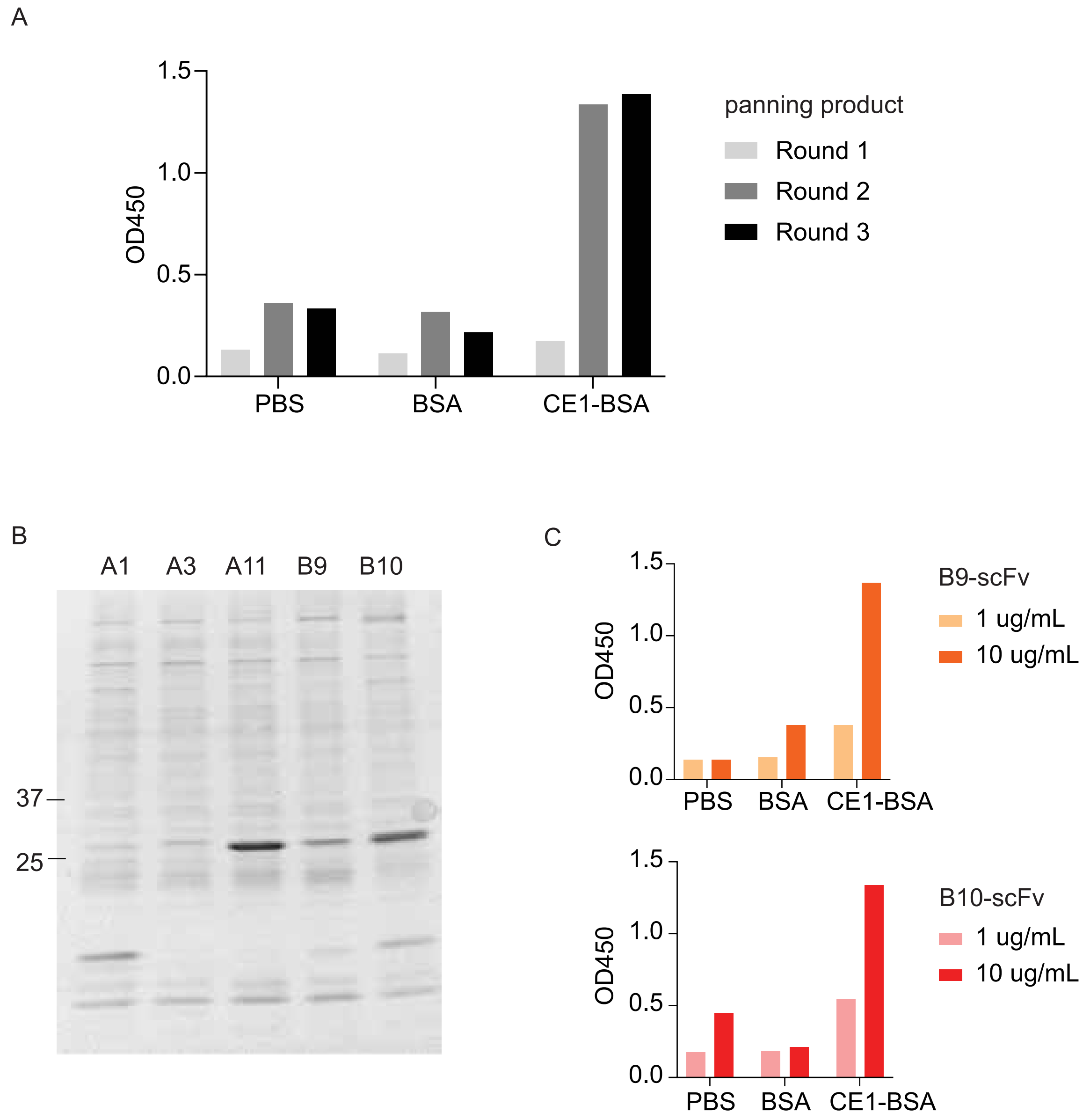

Extended Data Figure 2. Identification of CE1-reactiving scFv from phage-display human scFv library search.

(A)The binding between BSA-CE1-conjugated peptide and the product of the 1st, 2nd, and 3rd round of panning was measured using ELISA.

(B)Western blots of the five scFv candidates after recombinant expression.

(C)Dose-dependent CE1-binding of B9-scFv and B10-scFv.

Extended Data Figure 3

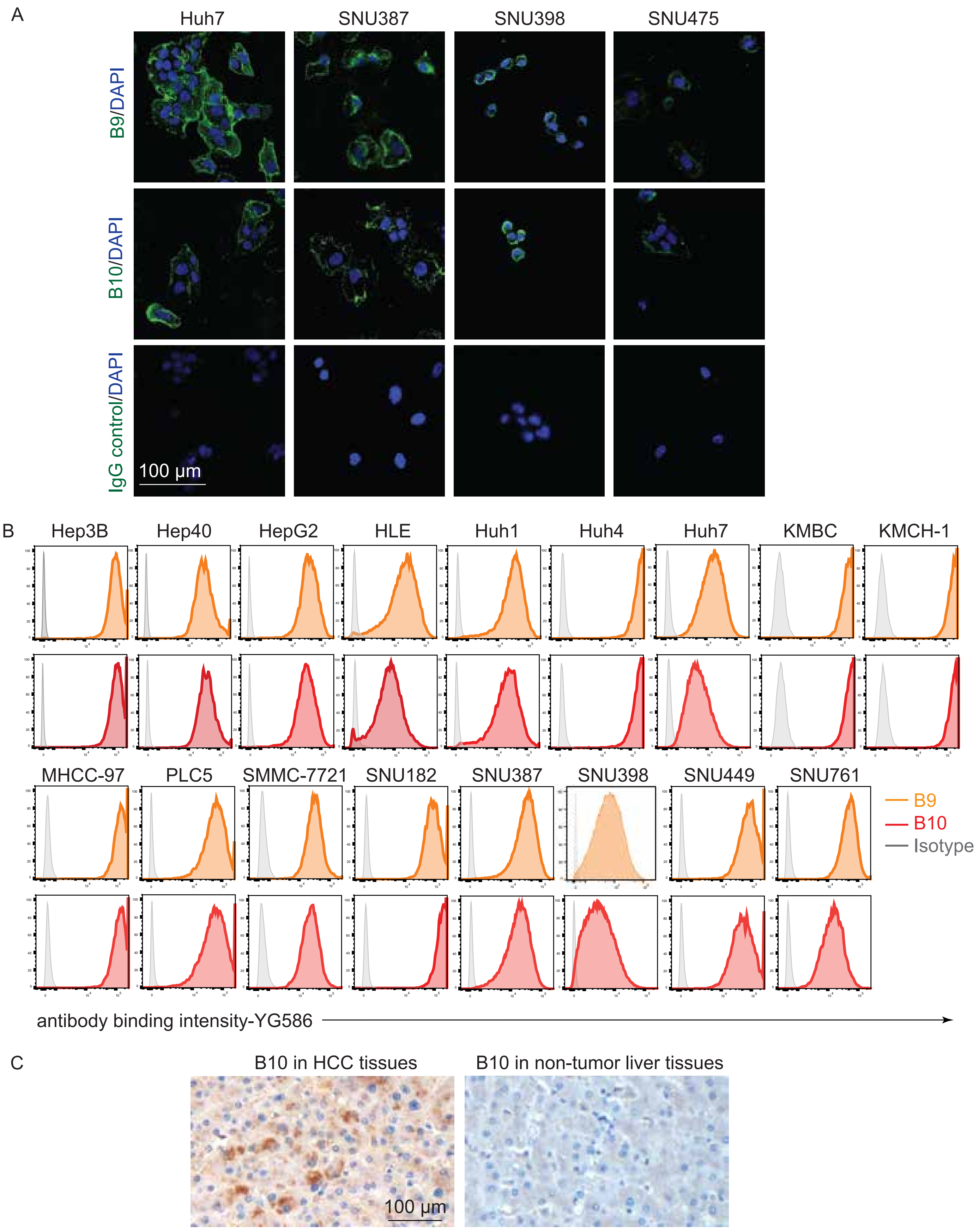

Extended Data Figure 3. Binding of B9 and B10 to human HCC cells and clinical HCC tissues.

(A) Representative images of immunofluorescence staining of B9, B10 and control antibody in Huh7, SNU387, SNU398, and SNU475 cells.

(B) Surface binding of B9, B10 and control antibody (2 μg/ml) to 17 HCC cell lines.

(C) Representative images of immunohistochemistry staining of B10 in clinical HCC tumor tissue and associated non-tumor tissue.

Extended Data Figure 4

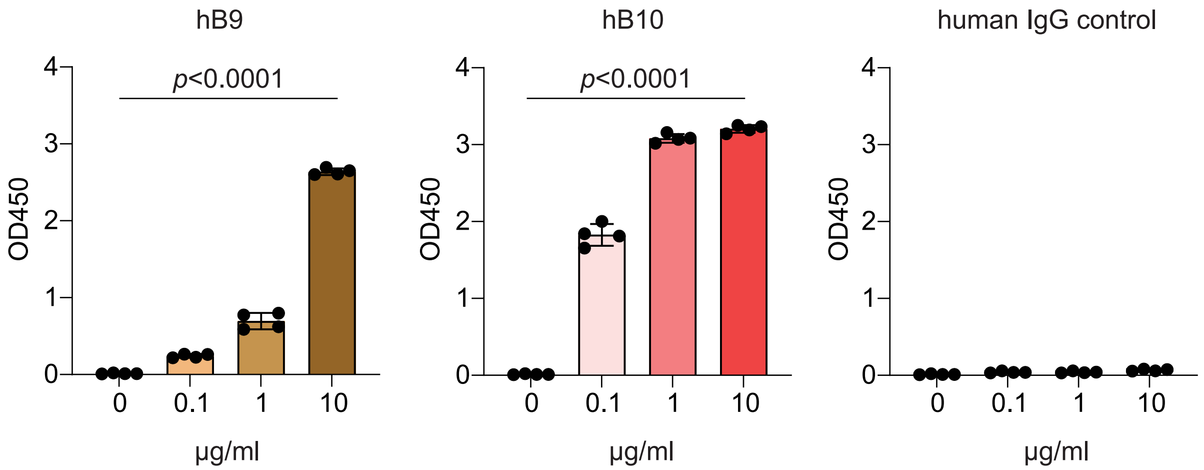

Extended Data Figure 4. Dose-dependent CE1-binding of hB9 and hB10.
Fully humanized B9- (hB9) and B10- (hB10) antibody and human IgG were incubated with CE1 at indicated doses for two hours and assessed by ELISA. Individual data points are plotted over mean bars with error bars representing ± standard deviation (SD). Statistical significance was determined using a one-way ANOVA test.

Extended Data Figure 5

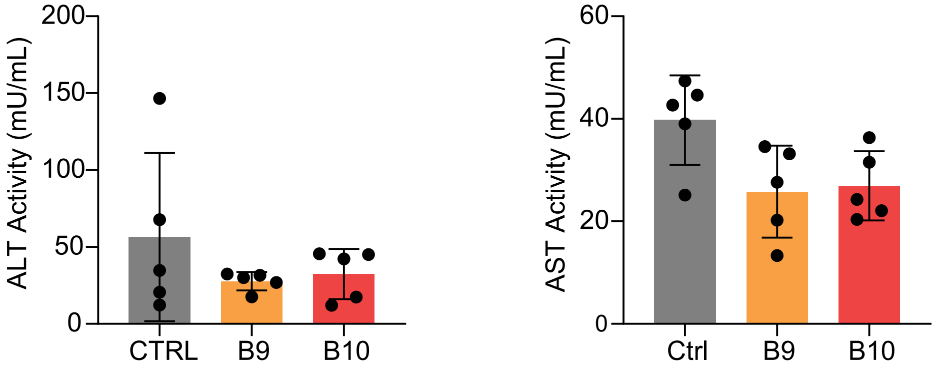

Extended Data Figure 5. Liver function of mice receiving B9, B10 and control antibody treatment.

Mice serums were collected upon completion of study for alanine aminotransferase and aspartate aminotransferase tests. (n=5) Individual data points are plotted over mean bars with error bars representing ± standard deviation (SD).

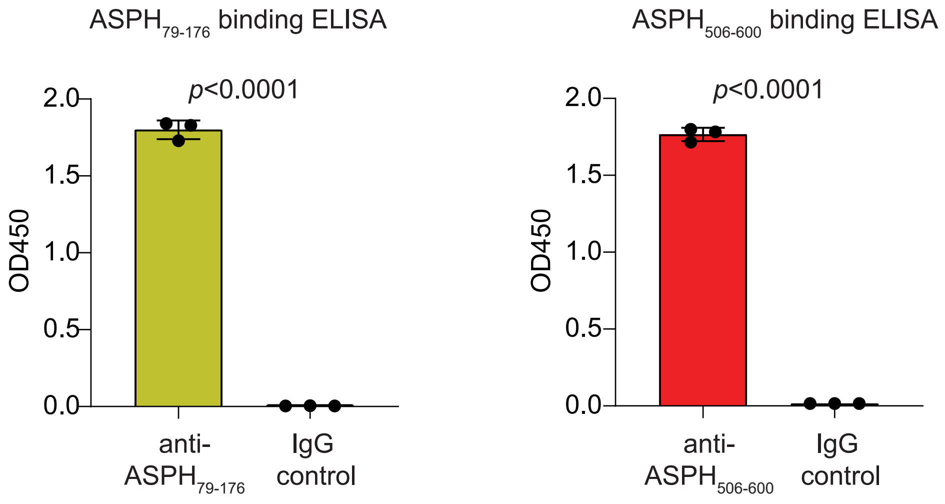

Extended Data Figure 6. Validation of the antigenicity of recombinant ASPH antigens. Recombinant ASPH79-176 and ASPH506-600 were incubated with the associated antibodies, namely anti- ASPH79-176 and anti-ASPH506-600, respectively, for two hours and assessed using ELISA. An isotype control was included for this experiment. (n=3) Individual data points are plotted over mean bar with error bars representing ± standard deviation (SD). Statistical significance was determined using a two-sided independent t-test.

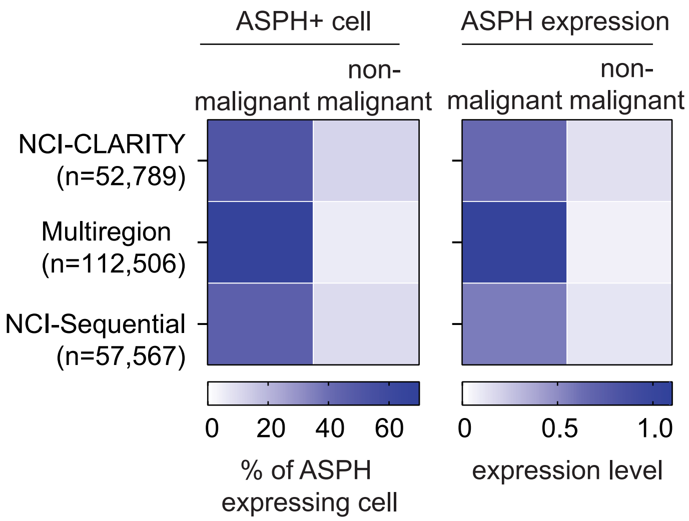

Extended Data Figure 7. Expression of ASPH in single cell transcriptomes associated with liver cancer cells and non-malignant cells.

Heatmap summarizes the presence of detectable ASPH and the level of ASPH expression in three independent single cell transcriptomic studies that include 31,780 malignant liver cancer cells and 157,820 non-malignant cells obtained from 101 patients.

Extended Data Table 1. Multivariate logistic regression analysis of viral serological activity and HCC diagnosis in TIGER-LC cohort.

Viral peptide	OR (95% CI)	P value
CE1-VP1	0.90 (0.8334-0.9730)	0.015381
Non-CE1 VP1	0.1528 (0.0141-1.6352)	0.121220
Non-VP1 EV/RV	0.5300 (0.0728-3.7813)	0.527815
Influenza	5.7638 (2.0754- 16.5734)	0.000928
Corona Virus	0.0020 (4.481486e-09 -314.2491)	0.323984

EV, enterovirus; RV, rhinovirus; OR, odds ratio; CI, confidence interval
